# Invasive Pneumococcal Isolates from Danish Infants (0 - 90 Days) during the Years 1943 to 2013

**DOI:** 10.1371/journal.pone.0106180

**Published:** 2014-08-26

**Authors:** Hans-Christian Slotved, Tine Dalby, Steen Hoffmann

**Affiliations:** Neisseria and Streptococcus Reference Laboratory (NSRlab), Department of Microbiology and Infection Control, Statens Serum Institut, Copenhagen, Denmark; Rockefeller University, United States of America

## Abstract

**Background:**

The seven-valent pneumococcal conjugate vaccine (PCV-7) was introduced in the Danish childhood immunization program (at 3, 5 and 12 months of age) in 2007 and was replaced with PCV-13 in 2010 without changes to the schedule. After the introduction of these vaccines the incidence of invasive pneumococcal disease (IPD) due to vaccine types (VTs) declined markedly in children aged 0–2 years; however, cases among infants too young to be protected by vaccination have not been studied in detail. We present data on IPD in infants less than 90 days from 1943 until 2013.

**Study design:**

The study included all infants younger than 90 days born from 1943 through 2013, who had not been PCV vaccinated and from whom a pneumococcus isolate from blood or cerebrospinal fluid had been submitted to the Danish national reference laboratory. All isolates were serotyped using Pneumotest Latex and Quellung reaction.

**Results:**

A total of 216 IPD cases were identified. The age group specific incidence (total number of IPD cases per 100,000 live births) varied from 0 to 16 in the period 1943 to 2007 and from 1.7 to 9.2 in the period 2008 to 2013. IPD cases due to PCV-7 serotypes were not observed later than 2009.

**Conclusion:**

In Danish infants younger than 90 days, IPD due to PCV-7 serotypes has decreased and has not been observed since 2009, but the total incidence of IPD has not changed.

## Introduction

*Streptococcus pneumoniae* (pneumococcus) is worldwide a cause of high morbidity and mortality among children under 5 years of age and among the elderly [1]. Invasive pneumococcal disease (IPD) is a frequent cause of bacteraemia and meningitis in infants in Denmark as well as globally [2], [3]. With the introduction of the pneumococcal conjugate vaccine (PCV) in children, an effective protection has been provided against IPD caused by the serotypes included in the vaccine [1], [4]. The first conjugate vaccine, PCV-7 (Pfizer Vaccines), was licensed in USA in 2000 [5] and covered seven different serotypes. The PCV-7 has since then been followed by the PCV-10 (GlaxoSmithKline Biologicals) and PCV-13 (Pfizer Vaccines) thereby expanding the vaccine coverage [1], [3] to 10 and 13 serotypes, respectively.

In Denmark, the PCV-7 was introduced into the Danish childhood immunization programme in October 2007 and is offered free of charge to all children at the age of 3, 5 and 12 months [6]. Furthermore, a catch-up programme was introduced for children younger than 17 months (born after April 2006) [7]. Healthy infants younger than 90 days are not vaccinated. In April 2010, the PCV-7 was replaced with PCV-13 [7], including six additional serotypes compared to PCV-7. The uptake of children in the PCV vaccination in Denmark is 90% depending for the first and second dose and 80% for the third dose [7]. An analysis of data on IPD cases from the last seven decades in Denmark showed that with the introduction of PCV-7 and PCV-13, the incidence of IPD cases due to vaccine serotypes decreased markedly [7], [8]. This decline among both vaccinated and non-vaccinated children was observed three years after the introduction of the PCV [5]. Also in other countries, the introduction of PCV has resulted in a decrease in IPD cases due to VTs among non-vaccinated individuals, i.e. herd protection, in particular among the elderly [9], [10]. Several studies have shown the effect of PCV on the reduction of VTs but also the appearance of pneumococcal serotypes not included in the PCV-vaccines [3], [5], [10]. In England and Wales [11] as well as Utah, USA [12] a herd protection effect was observed among infants below approximately 90 days of age, in that PCV-7 serotypes decreased significantly as a cause of IPD in this age group.

In Denmark, similar data on infants (<90 days) has not been presented previously. In this study we aim at presenting an account of the fluctuation of IPD and serotypes in IPD cases from Danish infants below 90 days of age over a period from 1943 to 2013. The indirect effect of the PCV vaccines on the pneumococcal serotype distribution and IPD cases in infants is also described.

## Materials and Methods

### Study population

Since 1937, Statens Serum Institut has serotyped pneumococcal isolates, as described in detail by Harboe et al [8]. From 1943 the first data are available on children less than 90 days old with IPD, including information on age, serotype and type of IPD. Data from before 1980 must be interpreted with some caution [8]; e.g., blood culture techniques were of lower standard then, and the number of available pneumococcal typing sera was lower than now. Since October 2007 it has been mandatory for diagnostic laboratories to submit all isolates causing IPD to Statens Serum Institut (SSI) for serotype identification and registration [13].

Eligible for entry in the present study were infants younger than 90 days who were not PCV vaccinated and who were registered in the laboratory database 1943–2013 with IPD, i.e. who had pneumococci identified in blood, cerebrospinal fluid or other usually sterile sites [8]. Each patient was represented by only one isolate in this study.

### Identification of pneumococcal isolates

The isolates were identified by optochin susceptibility and bile solubility tests. All isolates were serotyped either by Quellung reaction alone or by Pneumotest Latex, confirmed by Quellung reaction using type-specific pneumococcal rabbit-antisera (SSI-Diagnostica, Copenhagen, Denmark) as previously described [8], [14], [15].

### Data analysis

Data were analysed using Graph Pad Prism version 5 (GraphPad Software) for descriptive statistical analysis.

### Ethical considerations

The study was a retrospective, population-cohort study based on national laboratory surveillance data on IPD. Since data and samples from patients were collected routinely for diagnostic and national surveillance purposes, no ethical approval or informed consent from patients were required. The study was approved by the Danish Data Protection Agency (record number 2007-41-0229).

## Results

In total 216 infants were identified from 1943 to 2013 with IPD. All isolates were from blood or cerebrospinal fluid. The age of the infants ranged from 0 days to 89 days with a median age of 40 days. There were 96 females, 112 males and 8 with no information about gender. A total of 100 were diagnosed with bacteraemia and 116 with meningitis; no other types of invasive pneumococcal infection were found. For the period before the introduction of PCV-7 in 2007 (1943–2006), the median age of the infants was 39 days (range 0–89; n = 191) and in the period after the introduction of PCV-7 (2007–2013), the median age was 51 days (range 2–89; n = 25). The infants with IPD before 2006 was significant younger than the infants with IPD from 2007 (Mann-Whitney; P = 0.0421).

**Figure 1:**
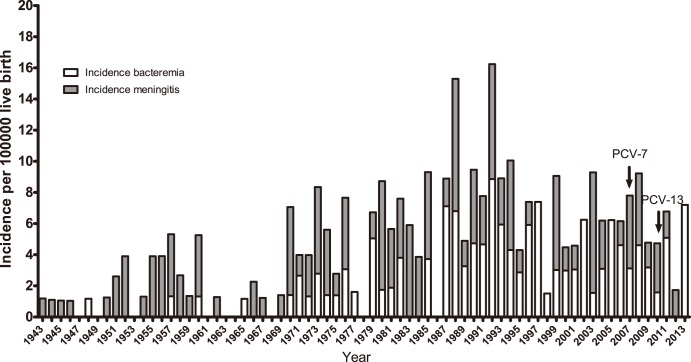
Incidence of IPD in infants younger than 90 days from 1943 through 2013. Grey bars: meningitis. White bars: bacteraemia. PCV-7 was introduced in October 2007 and PCV-13 was introduced in April 2010. The range of the incidence rate and Confidence Intervals (CI: 95%) for IPD was from 0–16.2 (lower CI: 0–9; upper CI 0–29.4), for meningitis 0–8.5 (lower CI: 0–3.5; upper CI 0–20.4) and for bacteraemia 0–8.9 (lower CI: 0–4.0; upper CI 0–19.7).

**Figure 2:**
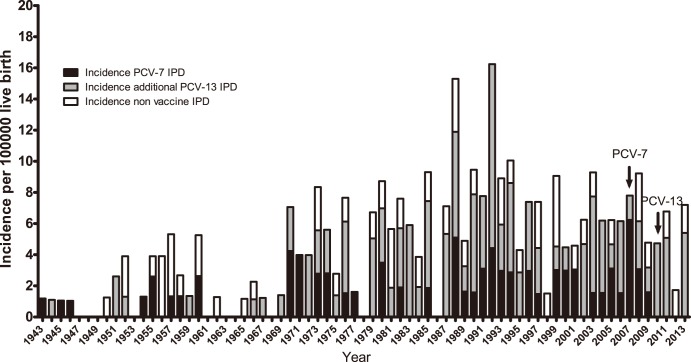
Incidence of IPD in infants younger than 90 days from 1943 through 2013. Black bars: serotypes included in the PCV-7. Grey bars: additional serotypes included in the PCV-13. White bars: serotypes not included in the PCV-13. PCV-7 was introduced in October 2007 and PCV-13 was introduced in April 2010. The range of the incidence rate and Confidence Intervals (CI: 95%) for PCV-7 was from 0–6.2 (lower CI: 0–2.3; upper CI 0–16.6), for PCV-13 0–11.8 (lower CI: 0–5.9; upper CI 0–23.6) and for non-PCV-13 0–4.5 (lower CI: 0–1.5; upper CI 0–15.1).

Figure 1 presents the incidence of cases of either bacteraemia or meningitis from 1943 until 2013. The incidence of cases of meningitis has been relatively stable since 1943, while that of bacteraemia increased from around 1970 and onwards. The number of IPD cases in infants has been within the range of 0 to 11 per year with a median of 3 (median incidence was 4.5). The range of the incidence rate and the 95% confidence intervals (CI) for IPD was 0–16.2 (lower CI: 0–9; upper CI 0–29.4), for meningitis 0–8.5 (lower CI: 0–3.5; upper CI 0–20.4) and for bacteraemia 0–8.9 (lower CI: 0–4.0; upper CI 0–19.7). From 2010 (Figure 2, Table 1), i.e., three years after the introduction of PCV-7, the corresponding PCV-7 VTs have not been observed among IPD cases in infants. The additional six VTs included in PCV-13 are, however, still observed. From 1943 to 2013, 17 different non-PCV-13 serotypes have been observed; however, since 1999 only serotypes 6C, 8, 12F, 20 and 27 have been observed (8 cases) (Table 1). The range of the incidence rate and confidence intervals (CI: 95%) for PCV-7 was 0–6.2 (lower CI: 0–2.3; upper CI 0–16.6), for PCV-13 it was 0–11.8 (lower CI: 0–5.9; upper CI 0–23.6) and for non-PCV-13 it was 0–4.5 (lower CI: 0–1.5; upper CI 0–15.1).

**Table 1:** Pneumococcal serotypes from infants younger than 90 days with invasive pneumococcal disease from 2003 through 2013.

Year	Number of isolates (Incidence, 95% CI)	PCV7 serotypes	PCV13 serotypes	Non-vaccine serotypes
**2003**	6 (9.2, 4.2–20.7)	23F	1, 1, 7F, 7F	8
**2004**	4 (6.2, 2.3–16.5)	19F	1,3, 7F	
**2005**	4 (6.2, 2.3–16.6)	18C, 18C	1	20
**2006**	4 (6.2, 2.3–16.4)	6B	1, 3,7F	
**October 2007: Introduction of PCV7 in the Danish childhood immunization program**				
**2007**	5 (7.8, 3.3–18.8)	18C, 19F, 19F, 23F	19A	
**2008**	6 (9.2, 4.1–20.5)	6B, 19F	7F, 7F	6C, 27
**2009**	3 (4.8, 1.5–14.8)	18C	7F	6C
**April 2010: Introduction of PCV13 in the Danish childhood immunization program**				
**2010**	3 (4.7, 1.5–14.7)		3, 7F, 19A	
**2011**	4 (6.8, 2.5–18.1)		1, 3, 7F	27
**2012**	1 (1.7, 0.2–12.3)			12F
**2013**	4 (7.2, 2.7–19.19)		1, 7F, 7F	8

**Figure 3:**
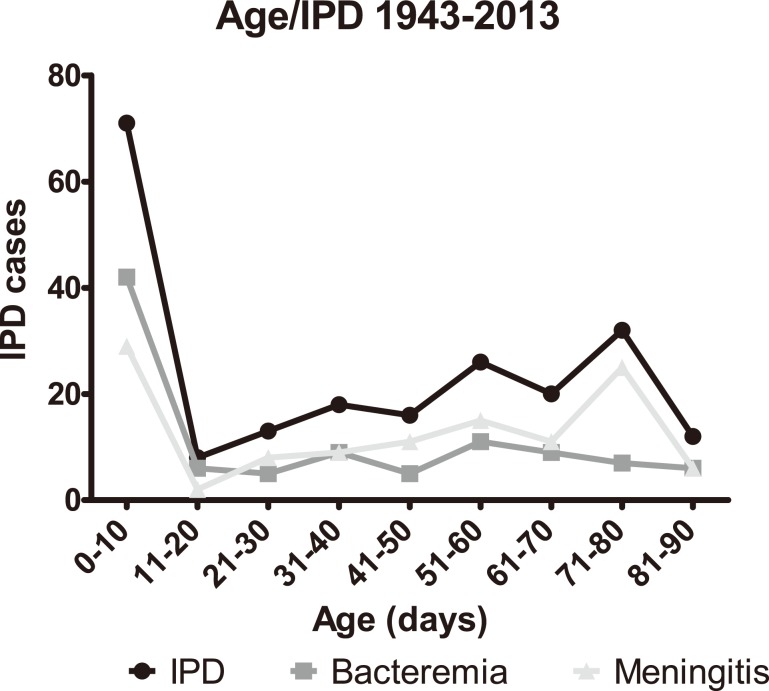
Number of cases of IPD (in total, meningitis, and bacteraemia) in children younger than 90 days from 1943 through 2013.

Infants aged 10 days or younger accounted for 33% of all IPD cases while 67% were among infants aged 11–90 days, with a tendency of increasing occurrence of IPD cases with age (Figure 3). Meningitis cases showed a similar pattern as all IPD cases, in that infants aged 0–10 days accounted for 25% of cases; however a non-significant increase in the 71-80-day-old group was observed 42% of all bacteraemia cases were found in infants 0–10 days, and the remaining cases were distributed rather evenly across the older age-groups.

**Figure 4:**
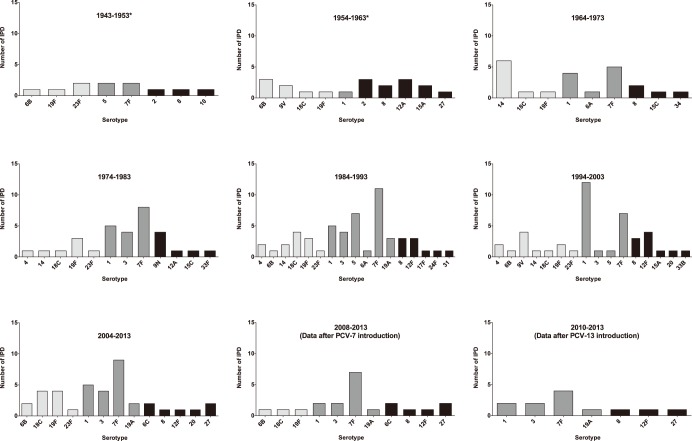
IPD serotypes observed in infants less than 90 days from 1943 through 2013. Light grey bars represent serotypes included in the PCV-7. Grey bars represent serotypes included in the PCV-13. Black bars represent non-vaccine serotypes. *Identification of isolates in the early years has to be considered with precaution.

Overall, serotypes 7F and 1 were the predominant serotypes found over the years, accounting for 20% and 15%, respectively, of all the IPD cases (Figure 4). Serotypes 19F (7%), 3 (6%), 18C (6%), and 8 (6%) were also very common serotypes. Serotype 8 has been observed repeatedly over the decades; however the majority of the other non-PCV-13 serotypes appeared only rarely.

## Discussion

It has been mandatory to submit pneumococcal isolates to the surveillance at Statens Serum Institut since October 2007 [13]. The coverage has been estimated to be 90 to 95% [8]. The representativeness of information from the early decades of our laboratory database has been reviewed by Harboe et al [8].

PCV-7 was introduced in the Danish childhood vaccination programme in October 2007, and from April 2010 it was replaced with PCV-13 [5]. The vaccination schedule is 3, 5, and 12 months, thus, infants below 90 days are not vaccinated. A herd protection effect caused by the PCV-7 has been demonstrated in this age group [11], [12]. These studies ended in 2010 and showed a decrease of IPD caused by PCV-7 serotypes, like our study. Both Olarte et al [12] and Ladhani et al [11] found that a majority of IPD cases in infants were due to the additional six serotypes included in the PCV-13. Therefore, they anticipated that a further decrease would occur after the introduction of PCV-13. However, our study which continued through 2013 could not confirm this hypothesis, although it is possible that a decrease or even a disappearance may take effect during the forthcoming years.

Findings regarding the overall incidence of IPD in infants younger than 90 days after PCV introduction are not congruent. Ladhani et al [11] detected a decrease in the overall incidence after the PCV-7 introduction. In contrast, no decrease was evident from the results obtained by Olarte et al [12], and neither from the present study, which included three additional years after the introduction of PCV-13.

Thus, the virtually unchanged incidence of IPD in Danish infants younger than 90 days was due to the additional PCV-13 serotypes and a few non-PCV-13 serotypes. The number of non-PCV-13 serotypes is too low to support a suspicion of type shift (Table 1). Olarte et al [12] found that the overall IPD incidence remained stable due to an increase of the six additional PCV-13 serotypes, i.e. non-PCV7-serotypes.

While PCV-7 VTs have not been isolated from IPD in this age group from 2010 onwards, it is still too early to ascertain when and if the prevalence of PCV-13 VTs in Denmark will diminish. However, both the fluctuation and incidence of IPD cases after the introduction of the PCVs might partly be due to normal variation in IPD cases over time, and not only an effect of the PCV introduction and herd protection (Figures 1 and 2).

In the present study, 33% (71/216) of the IPD cases occurred in 0-10-day- old infants (Figure 3). The finding of such cases of so-called early-onset IPD is in agreement with the study by Ladhani et al [11] who found that 40% of the patients (101/256) were 0–6 days old. The corresponding number in our study is 31% (67/216). In the study by Olarte et al [12], 33% (3/9) were below 30 days of age, but the total number was very low. With regards to the small peak of meningitis cases among the 71–80 day old infants in figure 3, then this was not significantly different compared to meningitis cases in infants from the groups of 61–70 or 81–90 days. Because of the limit data have we not been able to find an explanation for this peak of meningitis among the 71–80 day infants.

The study only describes the occurrence of IPD cases and the distribution of serotypes in these cases. No clinical data were available, and it is therefore a limitation of our study that that we could not evaluate neither possible differences in full-term compared to pre-term infants nor possible associations between underlying diseases and IPD outcome.

## Conclusion

In conclusion, in the three years following the introduction of PCV-7 in Denmark in 2007 the corresponding seven vaccine types have not been found as a cause of IPD in unvaccinated infants younger than 90 days. However, as non-vaccine types are gradually replacing vaccine types, the introduction of PCV-7 and PCV-13 in the Danish childhood vaccination programme have not yet had a significant impact on the total incidence of IPD cases in this age group.

## References

[1] Feldman C, Anderson R (2014) Review: Current and new generation pneumococcal vaccines. J Infect Jun 23 pii: S0163-4453(14)00166-2. doi: 10.1016/j.jinf.2014.06.006 10.1016/j.jinf.2014.06.006 24968238

[2] Howitz M, Hartvig Christiansen A, Harboe ZB, Mølbak K (2008) Surveillance of bacterial meningitis in children under 2 y of age in Denmark, 1997–2006. Scand J Infect Dis 40: 881–887. 18720256 10.1080/00365540802325914

[3] O’Brien KL (2013) PCV13 impact evaluations: the obvious and the unpredicted. Pediatr Infect Dis J. 32: 264–265. 10.1097/INF.0b013e3182787f89 23558322

[4] Borrow R, Heath PT, Siegrist CA (2012) Use of pneumococcal polysaccharide vaccine in children: what is the evidence? Curr Opin Infect Dis 25: 292–303. Review. 10.1097/QCO.0b013e3283531b0f 22517603

[5] Ingels H, Rasmussen J, Andersen PH, Harboe ZB, Glismann S, et al (2012) Impact of pneumococcal vaccination in Denmark during the first 3 years after PCV introduction in the childhood immunization programme. Vaccine 30: 3944–3950. 22504662 10.1016/j.vaccine.2012.03.060

[6] Harboe ZB, Valentiner-Branth P, Benfield TL, Christensen JJ, Andersen PH, et al (2010) Early effectiveness of heptavalent conjugate pneumococcal vaccination on invasive pneumococcal disease after the introduction in the Danish Childhood Immunization Programme. Vaccine 28: 2642–2647. 20096392 10.1016/j.vaccine.2010.01.017

[7] Harboe ZB, Valentiner-Branth P, Ingels H, Rasmussen JN, Andersen PH, et al (2013) Pediatric invasive pneumococcal disease caused by vaccine serotypes following the introduction of conjugate vaccination in Denmark. PLoS One 8: e51460. 23365635 10.1371/journal.pone.0051460 PMC3554759

[8] Harboe ZB, Benfield TL, Valentiner-Branth P, Hjuler T, Lambertsen L, et al (2010) Temporal trends in invasive pneumococcal disease and pneumococcal serotypes over 7 decades. Clin Infect Dis 50: 329–337. 20047478 10.1086/649872

[9] Rashid H, Khandaker G, Booy R (2012) Vaccination and herd immunity: what more do we know? Curr Opin Infect Dis 25: 243–249. 22561998 10.1097/QCO.0b013e328352f727

[10] Simell B, Auranen K, Käyhty H, Goldblatt D, Dagan R, et al. (2012) The fundamental link between pneumococcal carriage and disease. Expert Rev Vaccines11: 841–855. Review. 10.1586/erv.12.53 22913260

[11] Ladhani SN, Andrews NJ, Waight P, Borrow R, Slack MP, et al (2013) Impact of the 7-valent pneumococcal conjugate vaccine on invasive pneumococcal disease in infants younger than 90 days in England and Wales. Clin Infect Dis 2013 56: 633–40. 10.1093/cid/cis934 23175560

[12] Olarte L, Ampofo K, Stockmann C, Mason EO, Daly JA, et al (2013) Invasive Pneumococcal Disease in Infants Younger Than 90 Days Before and After Introduction of PCV7. Pediatrics 132: e17–24. 23733800 10.1542/peds.2012-3900 PMC3691535

[13] (2007) Bekendtgørelse om ændring af bekendtgørelse om lægers anmeldelse af smitsomme sygdomme m.v. (Executive Order no. 1102 of 20/9/2007). Sundhedsstyrelsen, den 20. September 2007.

[14] Kaltoft MS, Skov Sørensen UB, Slotved H-C, Konradsen HB (2008) An easy method for detection of nasopharyngeal carriage of multiple *Streptococcus pneumoniae* serotypes. J Microbiol Methods 75: 540–544. 18801391 10.1016/j.mimet.2008.08.010

[15] Lambertsen L, Kerrn MB (2010) Test of a novel *Streptococcus pneumoniae* serotype 6C type specific polyclonal antiserum (factor antiserum 6d) and characterisation of serotype 6C isolates in Denmark. BMC Infect Dis 10: 282. 20868480 10.1186/1471-2334-10-282 PMC2949764

